# Identification of cuproptosis-related lncRNAs to predict prognosis and immune infiltration characteristics in alimentary tract malignancies

**DOI:** 10.1186/s12859-023-05314-z

**Published:** 2023-05-04

**Authors:** Yangyang Xie, Xue Song, Danwei Du, Zhongkai Ni, Hai Huang

**Affiliations:** 1 Department of General Surgery, Hangzhou TCM Hospital Affiliated to Zhejiang Chinese Medical University, #453, Tiyuchang Road, Xihu District, Hangzhou, 310000 Zhejiang Province China; 2 Department of Pneumology, Hangzhou TCM Hospital Affiliated to Zhejiang Chinese Medical University, Hangzhou, 310000 Zhejiang Province China

**Keywords:** Cuproptosis, Long non-coding RNA, Prognosis, Immune infiltration, Alimentary tract malignancies

## Abstract

**Background:**

Alimentary tract malignancies (ATM) caused nearly one-third of all tumor-related death. Cuproptosis is a newly identified cell death pattern. The role of cuproptosis-associated lncRNAs in ATM is unknown.

**Method:**

Data from The Cancer Genome Atlas (TCGA) and Gene Expression Omnibus (GEO) databases were used to identify prognostic lncRNAs by Cox regression and LASSO. Then a predictive nomogram was constructed based on seven prognostic lncRNAs. In addition, the prognostic potential of the seven-lncRNA signature was verified via survival analysis, the receiver operating characteristic (ROC) curve, calibration curve, and clinicopathologic characteristics correlation analysis. Furthermore, we explored the associations between the signature risk score and immune landscape, and somatic gene mutation.

**Results:**

We identified 1211 cuproptosis-related lncRNAs and seven survival-related lncRNAs. Patients were categorized into high-risk and low-risk groups with significantly different prognoses. ROC and calibration curve confirmed the good prediction capability of the risk model and nomogram. Somatic mutations between the two groups were compared. We also found that patients in the two groups responded differently to immune checkpoint inhibitors and immunotherapy.

**Conclusion:**

The proposed novel seven lncRNAs nomogram could predict prognosis and guide treatment of ATM. Further research was required to validate the nomogram.

**Supplementary Information:**

The online version contains supplementary material available at 10.1186/s12859-023-05314-z.

## Introduction

Alimentary tract malignancies (ATM), comprising a spectrum of cancers occurring in the digestive tract, have seriously endangered public health and human life [1]. Arnold et al. reported the mortality of gastric (approximately 1.0 million new cases in 2018), esophagus (570,000 cases), and colorectum (1.8 million cases) cancer, which caused nearly one-third of all tumor-related deaths [2]. The alimentary tract, from the oropharynx to the anal canal, is closely related in mainly organ functions and development, suggesting common etiological pathways and mechanisms. However, the mechanisms of digestive tract tumorigenicity in common remain to be explored.

There is currently no efficient and established early-stage screening protocol for ATM patients. As a result, many patients are in the middle and advanced stages when they are diagnosed [2]. The average survival duration of individuals with advanced ATM continues to be extremely low, despite advancements in several therapy approaches. Therefore, it is essential to explore a reliable prognostic signature to predict the prognosis of ATM patients and direct clinical practice.

According to Science, a novel type of cell death known as cuproptosis is brought on by an excessive buildup of copper. Copper causes toxic protein stress and, as a result, cell death by binding specifically to lipoylated parts of the tricarboxylic acid (TCA) cycle [3]. A trace element called copper is essential for many biological activities. Key elements of the course of cancer, including angiogenesis, metastasis, and proliferation, are influenced by copper buildup [4, 5]. Growing research over the past few years has demonstrated that copper homeostasis dysregulation may influence the onset and development of ATM. Jacinta et al. demonstrated that copper played a significant part in the biological activity that was seen to be amplified after being associated with a lipid-based nanosystem for the treatment of colorectal cancer [6]. Rebecca et al. used a multi-technology strategy to examine the mechanism of sensitivity in esophageal cancer to copper-dependent cell death, which could be targeted in the future [7]. In another study, Du et al. presented that disulfiram (DSF) was highly toxic to gastric cancer cells in a copper-dependent manner. And DSF/Cu exerted antitumor activity against gastric cancer cells in vitro and in vivo [8].

Long non-coding RNAs (lncRNAs) are a subset of untranslated RNAs that comprise more than 200 nucleotides [9]. Growing data reveals that lncRNAs have the capacity to control tumor metastasis, cancer immunity, and programmed cell death in recent years [10–13]. Additionally, fresh possible prognostic markers known as lncRNAs have been discovered for ATM patients [14, 15]. Sun et al. explored the lncRNA-mRNA regulatory networks in ATM progression and performed bioinformatic analysis, indicating that THBS2 was a potential key regulator and therapeutic target [16]. Hu et al. reported that lncRNA EGFR-AS1 could regulate the expression of EGFR via heightening EGFR mRNA stability to active phosphatidylinositol-3 kinase (PI3K)/AKT pathway for the furtherance of the malignant progression of ATM [17]. Besides, Hao et al. identified immune-related lncRNA pairs and constructed a predictive nomogram, which demonstrated good predictive ability [18]. Nevertheless, research on cuproptosis-related lncRNAs in ATM is still limited.

Given that integrated analyses usually emphasized cancer heterogenicity, this study tried another view of homogeneity of cancer prognosis, immunological landscape, and somatic gene mutation. By using bioinformatics analysis, we identified novel cuproptosis-related risk lncRNAs and constructed a predictive model of digestive tract cancers, which could eventually guide doctors to make better clinical decisions.

## Materials and methods

### TCGA and GEO data

We downloaded RNA sequencing (RNA-seq), expression files, and mutation files from the Cancer Genome Atlas (TCGA) database (https://portal.gdc.cancer.gov/repository), including 375 tumor samples and 32 normal samples in STAD, 163 tumor samples and 11 normal samples in ESCA, 480 tumor samples and 41 normal samples in COAD, and 167 tumor samples and 10 normal samples in READ. These data were arranged in accordance with TCGA procedures and combined into transcripts per million data (TPM). GSE40967, GSE53622, and GSE84437 from the Gene Expression Omnibus (GEO) database (https://www.ncbi.nlm.nih.gov/geo/) were downloaded and used as an external validation cohort.

### Identification of cuproptosis-related lncRNAs

A list of 16 cuproptosis regulators was retrieved from the lipoylated TCA cycle pathway of copper-induced cell death (FDX1, LIPTI, LIAS, DLD, MTF1, GLS, CDKN2A, DLAT, PDHA1, PDHB, DBT, GCSH, and DLST) [3] and copper transport protein (SLC31A1, ATP7A, and ATP7B) [19, 20].

The co-expression analysis between lncRNAs and cuproptosis-related genes was then carried out (|cor|> 0.4 and *p* < 0.001). The "DEseq2" package was used to identify lncRNAs that were differentially expressed between tumor and normal samples. The following requirements were met by differentially expressed lncRNAs: *p* < 0.05 and (log2FC|> 1 (FC, fold change). A Sankey diagram was mapped to assess the degree of association between genes involved in cuproptosis and lncRNAs.

### Cuproptosis-related lncRNAs signature for ATM prognosis

To create and validate the cuproptosis-associated lncRNAs signatures, the overall patients were randomly divided into either the training cohort or the test cohort in a 7:3 ratio. Univariate Cox regression analysis was initially applied to identify the risk lncRNAs. The LASSO regression analysis based on tenfold cross-validation was then used to reduce the overfitting effect. Finally, the multivariate Cox regression analysis was used to identify the best prognostic signs.

We then created a risk score algorithm for ATM by calculating the relevant coefficients for the risk lncRNAs. The risk score for each patient was calculated using the following formula: Risk score = ∑ coefi*αi, where αi and coefi denoted the expression level of each prognostic lncRNA and its accompanying coefficient.

### Validation of the lncRNA risk model

Based on the median risk score cutoff, patients in each cohort were divided into low-risk and high-risk groups. Using the "survminer" R package, the Kaplan–Meier survival analysis was used to compare the overall survival (OS) between two risk cohorts. The receiver operating characteristic (ROC) curve was constructed to evaluate the prognostic accuracy of our risk signatures. A principal component analysis (PCA) analysis was used to examine the distribution of high-risk and low-risk groupings. The prognostic signatures were analyzed both within the testing cohort and across the full cohort to determine the model's viability. The risk score and clinicopathologic parameters were subjected to univariate and multivariate Cox regression analysis to ascertain the independence of the cuproptosis-related lncRNAs signature (gender, age, grade, and TNM stage). To determine whether the signature maintained its predictive power in patient subgroups, stratified analysis was performed in the end.

### Establishment and assessment of the nomogram

The signatures and clinical data were incorporated into the suggested prediction nomogram. Then, ROC curves were employed to assess the predictive ability of the nomogram at 1-, 3-, and 5-year. The 1-, 3-, and 5-year calibration plots (1000 bootstrap resamples) were displayed to compare the projected overall survival with what was observed in the research. The 45-degree line was shown to be the best prediction.

### Functional and pathway enrichment

The "limma" package was used to find the differentially expressed genes (DEGs) between low- and high-risk groups. The following requirements were met by DEGs: false discovery rate (FDR) < 0.05 and |log2FC|≥ 1. The "clusterProfiler" package was used to conduct functional and pathway enrichment studies using the Gene Ontology (GO) and Kyoto Encyclopedia of Genes and Genomes (KEGG) databases [21]. Additionally, the top six important pathways in the high-risk and low-risk subgroups were found using the gene set enrichment analysis (GSEA) [22].

### Immunoassay

We used seven algorithms to investigate the relationship between the risk score and tumor-infiltrating immune cells, namely, CIBERSORT [23], CIBERSORT-ABS [23], EPIC [24], MCPcounter [25], quanTIseq [26], TIMER [27], and xCell [28]. The normalized enrichment score (NES) was utilized to produce individual enrichment scores for immunological pathways using single-sample GSEA (ssGSEA). The level of coordinated regulation of the genes within a sample was indicated by each ssGSEA enrichment score. Next, the immune checkpoint gene expression was investigated. Finally, the tumor immune dysfunction and exclusion (TIDE) algorithm was used to detect T cell malfunction signatures and signatures that excluded T cell infiltration into tumors to predict the therapeutic response of immune checkpoint blockades (ICBs) [29].

### Tumor mutation burden

Additionally, the TCGA Somatic Mutation Database was used to retrieve the data on somatic mutations for the ATM samples. We examined the tumor mutational burden (TMB) in both groups using the "maftools" package. The whole population was separated into high-TMB and low-TMB subsets by median TMB, and the Kaplan–Meier survival curve was presented for each group.

### Statistical analyses

R software (version 4.2.1) and Strawberry Perl (version 5.3.1) were used to perform all statistical analyses. *P* < 0.05 was regarded as statistical significance.

## Results

### Identification of cuproptosis-regulated lncRNAs

**Fig. 1:**
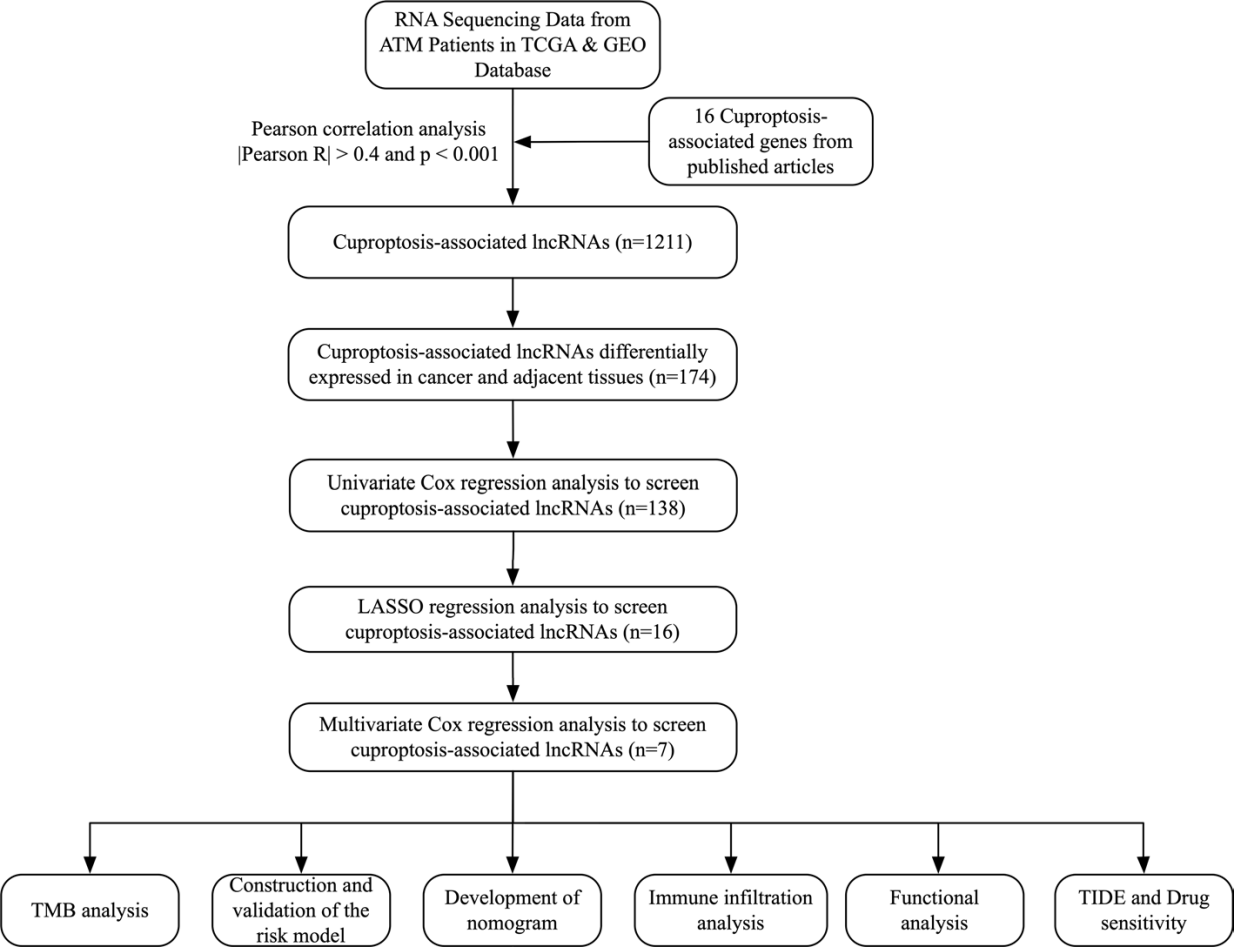
The process of the study

**Fig. 2:**
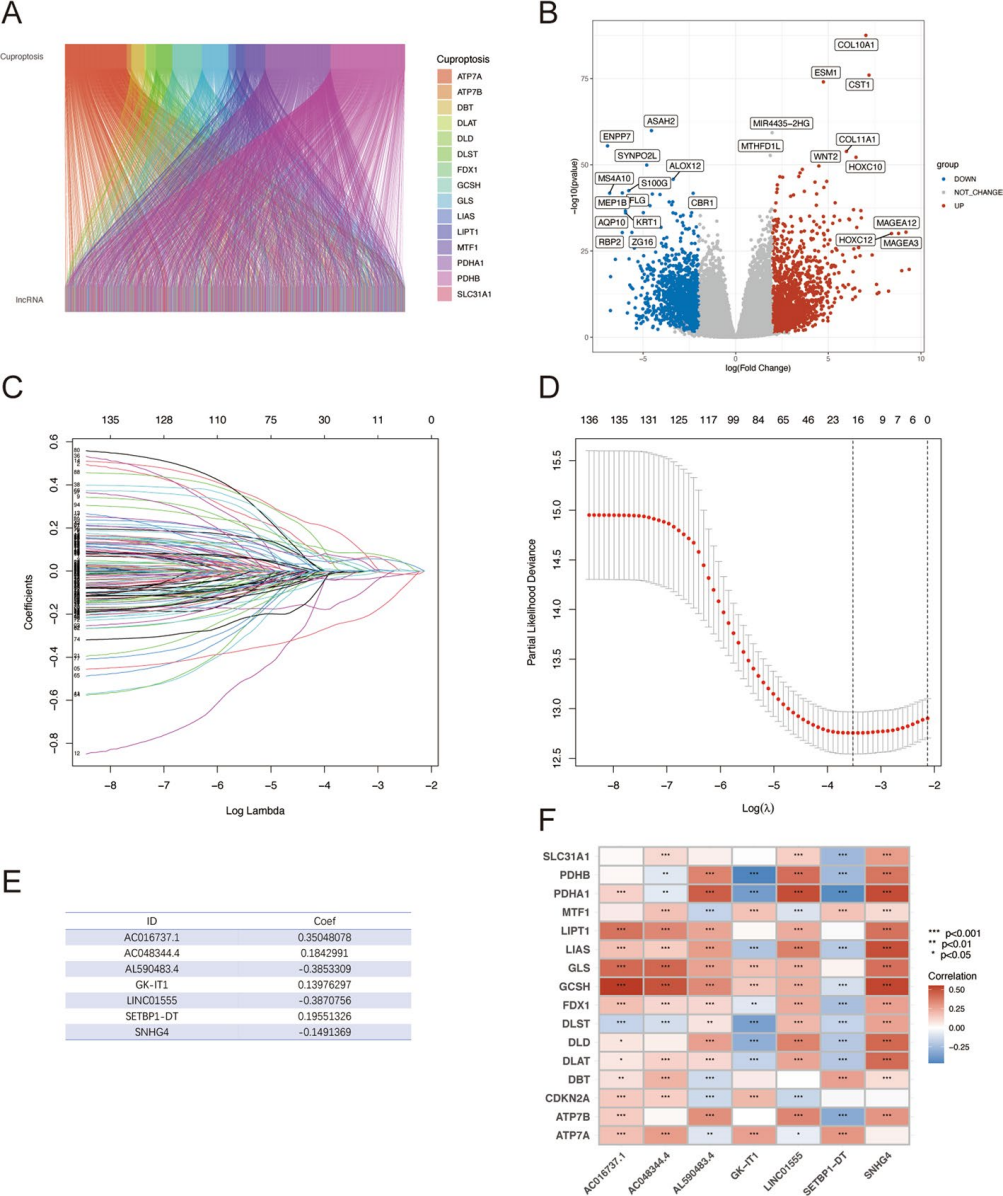
Identification of prognostic cuproptosis-related lncRNAs in ATM. **A** The Sankey diagram of the relationship between cuproptosis-related lncRNAs and genes. **B** The volcano plot of differentially expressed cuproptosis-related lncRNAs between normal and cancer samples. **C**, **D** LASSO regression algorithm identified the risk model. **E** The seven risk lncRNAs prognostic signature. **F** Correlation between cuproptosis-associated genes and risk lncRNAs in the TCGA-ATM cohort. Each unit's color indicated the level of association. *p < 0.05, **p < 0.01, and ***p < 0.001

The workflow of this project was presented in Fig. 1. From the TCGA database, RNA-sequencing information and clinical annotation for ATM were retrieved. In a recent publication, 16 cuproptosis regulators were identified in the lipoylated TCA cycle pathway of copper-induced cell death (Additional file 1: Table S1). The Ensembl gene annotation dataset discovered 16,901 lncRNAs and 19,962 mRNAs altogether. Based on the Pearson correlation analysis (|cor|> 0.4 and *p* < 0.001), 1211 cuproptosis-related lncRNAs were discovered. The co-expression network between the cuproptosis-associated lncRNAs and cuproptosis genes was then presented by the Sankey plot (Fig. 2A). Then, using the criterion of *p* < 0.05 and (log2FC|> 1, 174 differentially expressed lncRNAs between normal and cancer samples were chosen for additional study (Fig. 2B). 118 of these genes showed an upregulation, while 56 showed a downregulation.

### Risk model

We randomly divided all ATM cases into the training set and the internal validation set at a 7:3 ratio. The training set was used to create the model, and the internal validation set was used to validate the model. The chi-square test revealed that the demographic and clinicopathologic characteristics of the two groups were comparable (Table 1). First, 138 lncRNAs that were significantly linked with the OS (*p* < 0.05) were originally screened using a univariate Cox proportional hazard regression analysis. Then 16 lncRNAs were retrieved by LASSO regression analysis (Fig. 2C, D). Finally, using the multivariate Cox regression model analysis, seven lncRNAs (AC016737.1, AC048344.4, AL590483.4, GK-IT1, LINC01555, SETBP1-DT, and SNHG4) were obtained for creating the ideal prognostic signature (Fig. 2E). The risk score was calculated as follows: risk score = (0.3505 × AC016737.1 expression) + (0.1843 × AC048344.4 expression) + (− 0.3853 × AL590483.4 expression) + (0.1398 × GK-IT1 expression) + (− 0.3871 × LINC01555 expression) + (0.1955 × SETBP1-DT expression) + (− 0.1491 × SNHG4 expression). The heatmap showed a close correlation between the seven risk lncRNAs and cuproptosis genes (Fig. 2F).

**Table 1:** The basic characteristics of ATM patients in the training and validation groups

Characteristics	All	Training	Validation	*P* value
	N = 1154	N = 808	N = 346	
*CancerType*				0.195
COAD	455 (39.4%)	331 (41.0%)	124 (35.8%)	
ESCA	163 (14.1%)	106 (13.1%)	57 (16.5%)	
READ	166 (14.4%)	110 (13.6%)	56 (16.2%)	
STAD	370 (32.1%)	261 (32.3%)	109 (31.5%)	
*Age*				0.264
≤ 65	535 (46.5%)	365 (45.3%)	170 (49.1%)	
> 65	616 (53.5%)	440 (54.7%)	176 (50.9%)	
*Gender*				0.466
FEMALE	447 (38.7%)	319 (39.5%)	128 (37.0%)	
MALE	707 (61.3%)	489 (60.5%)	218 (63.0%)	
*Grade*				0.595
G1	26 (5.3%)	17 (5.1%)	9 (5.8%)	
G2	200 (41.0%)	132 (39.6%)	68 (43.9%)	
G3	262 (53.7%)	184 (55.3%)	78 (50.3%)	
*Stage*				0.388
I	171 (15.7%)	112 (14.6%)	59 (18.4%)	
II	406 (37.3%)	285 (37.1%)	121 (37.7%)	
III	378 (34.7%)	274 (35.7%)	104 (32.4%)	
IV	134 (12.3%)	97 (12.6%)	37 (11.5%)	
*T*				0.072
T1	65 (5.8%)	48 (6.1%)	17 (5.1%)	
T2	220 (19.6%)	139 (17.6%)	81 (24.3%)	
T3	667 (59.3%)	477 (60.3%)	190 (56.9%)	
T4	173 (15.4%)	127 (16.1%)	46 (13.8%)	
*N*				0.467
N0	524 (47.1%)	361 (46.0%)	163 (49.5%)	
N1	310 (27.9%)	226 (28.8%)	84 (25.5%)	
N2	199 (17.9%)	137 (17.5%)	62 (18.8%)	
N3	80 (7.2%)	60 (7.7%)	20 (6.1%)	
*M*				0.362
M0	906 (88.3%)	631 (87.6%)	275 (89.9%)	
M1	120 (11.7%)	89 (12.4%)	31 (10.1%)	

**Fig. 3:**
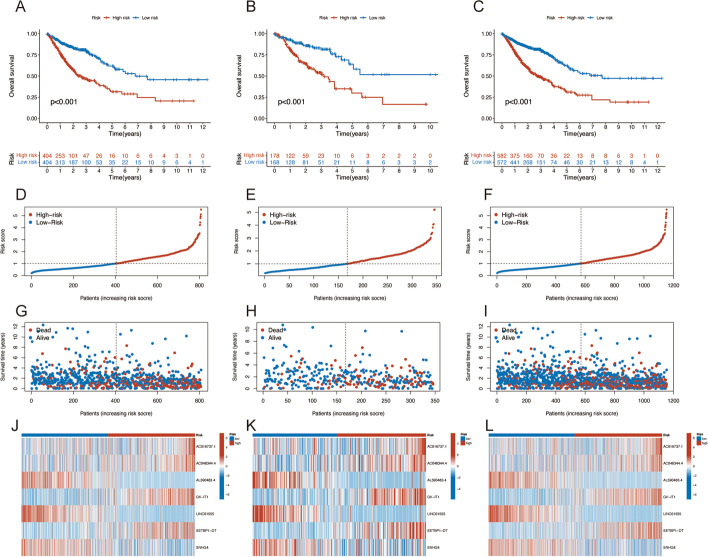
Prognosis capability of the model in the three patient sets. Kaplan–Meier survival analysis for OS (**A**–**C**), risk score distribution (**D**–**F**), the OS statuses (**G**–**I**), and heat maps of the seven CRLs (**J**–**L**) of the high-risk and low-risk cohorts in the training, testing, and entire subsets

In the training, internal validation, and overall sets, the samples were regrouped into high-risk and low-risk groups based on the median risk scores (Fig. 3). The distribution of risk scores, survival time patterns, survival status, and the associated expression of seven risk lncRNAs were validated in the three groups. For all three analysis groups, the same trend results were observed. The Kaplan–Meier curves showed better survival in the low-risk group than in the high-risk group (all *p* < 0.001) (Fig. 3A–C). The risk curves and scatterplots showed that samples in high-risk groups had significantly higher mortality rates (Fig. 3D–I). The heatmap showed that three protective lncRNAs were noticeably downregulated whereas four risk lncRNAs were noticeably increased in the high-risk group (Fig. 3J–L). These all suggested that the risk prediction model demonstrated a good capacity for prediction.

### Assessment of the risk model

**Fig. 4:**
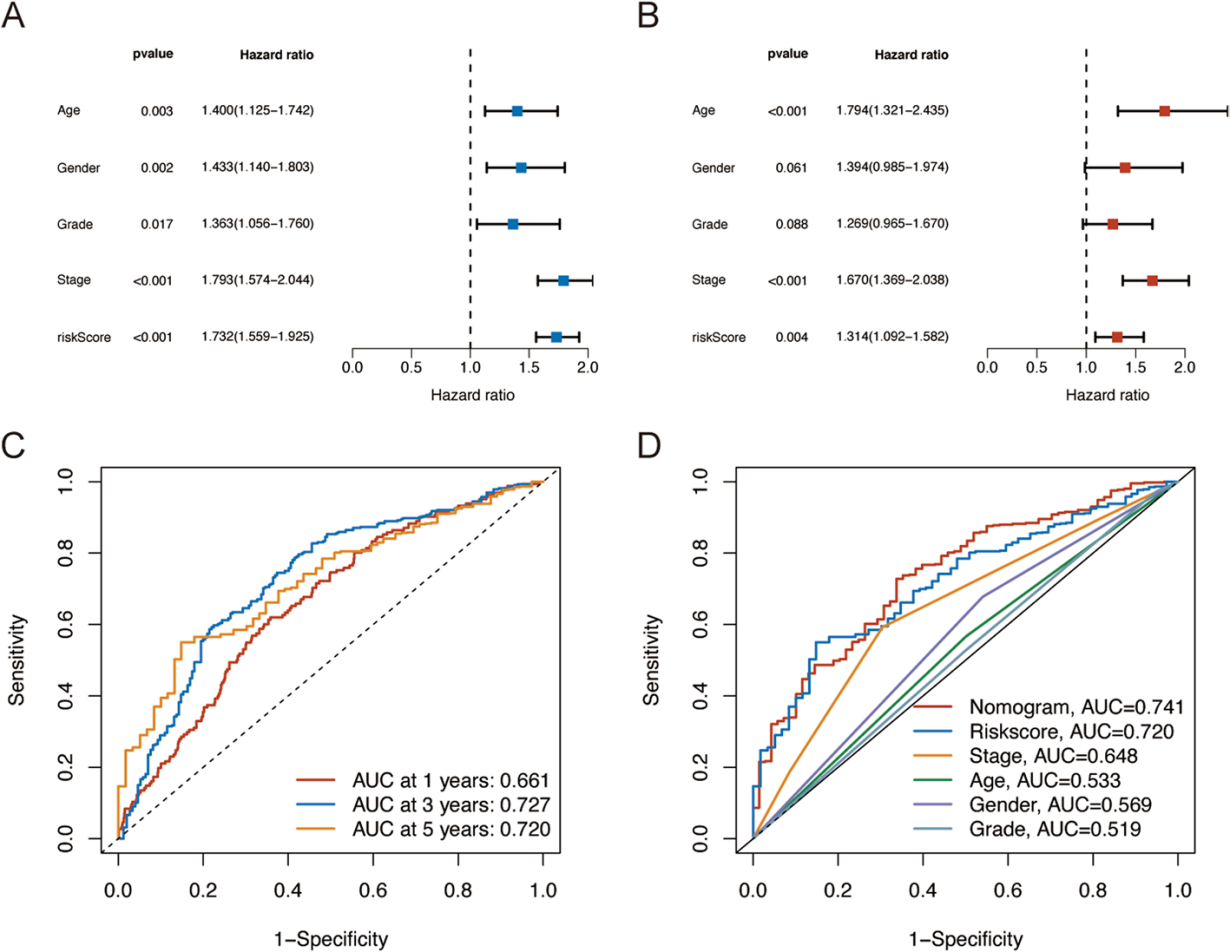
Validation of the model. **A**, **B** Uni-Cox and multi-Cox analyses of OS for risk score, gender, age, stage, and grade. **C** Time-dependent ROC curve analyses of the risk score model at 1-, 3-, and 5-year. **D** Comparison of the ROC curves of the nomogram, risk score, gender, age, stage, and grade

To determine if the risk score may function as an independent prognostic factor for ATM, univariate and multivariate Cox regression analyses were used. Age, gender, grade, stage, and risk score were all positively correlated with the prognosis of ATM in the entire sample, according to the results of the univariate Cox regression analysis (*p* < 0.001). (Fig. 4A; Additional file 2: Table S2). Age, stage, and risk score were also shown to be independent prognostic factors in ATM by the multivariate Cox regression analysis (*p* < 0.05) (Fig. 4B; Additional file 2: Table S2). In the univariate and multivariate analyses, the HR values for risk scores were 1.732 (1.559–1.925) and 1.314 (1.092–1.582).

The predictive power of the risk signature for the OS was evaluated using the ROC curve. At 1-, 3-, and 5-year, this risk score's predictive ability was strong (Fig. 4C). When compared to other clinicopathologic variables, the AUC associated with the risk score was the highest (Fig. 4D). Additionally, we investigated how the risk model affected various subgroups using Kaplan–Meier curves, which showed good performance in most subgroups (*p* < 0.05) (Additional file 5: Fig. S1). Above all, our findings showed that the risk score based on the signatures of the seven risk lncRNAs was useful for prognostic analysis.

### Nomogram

**Fig. 5:**
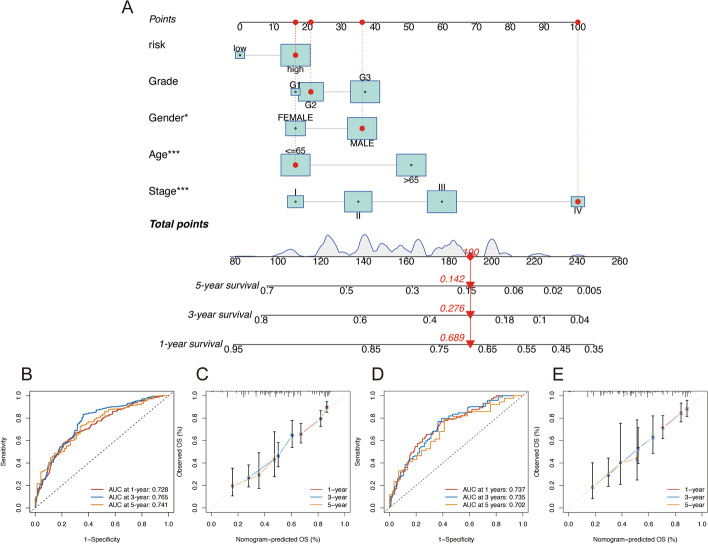
**A** Nomogram for survival prediction. **B**, **C** The ROC curves and calibration curves in the training cohort. **D**, **E** The ROC curves and calibration curves in the external validation cohort

We created a nomogram based on the training cohort to compute the overall survival rate of 1-, 3-, and 5-year using the risk score in combination with clinicopathological variables such as age, gender, grade, and stage (Fig. 5A). The nomogram's AUC values for the 1-, 3-, and 5-year periods were 0.728, 0.765, and 0.741, respectively, demonstrating its strong predictive power (Fig. 5B). The calibration plots showed good agreement for the accuracy of 1-, 3-, and 5-year overall survival prediction (Fig. 5C). The nomogram displayed a greater AUC compared to the single parameter in the model. Then the model was verified by using samples from the GEO cohort. The results indicated ideal predictive ability in external validation (Fig. 5D, E).

### PCA and biological pathways analyses

**Fig. 6:**
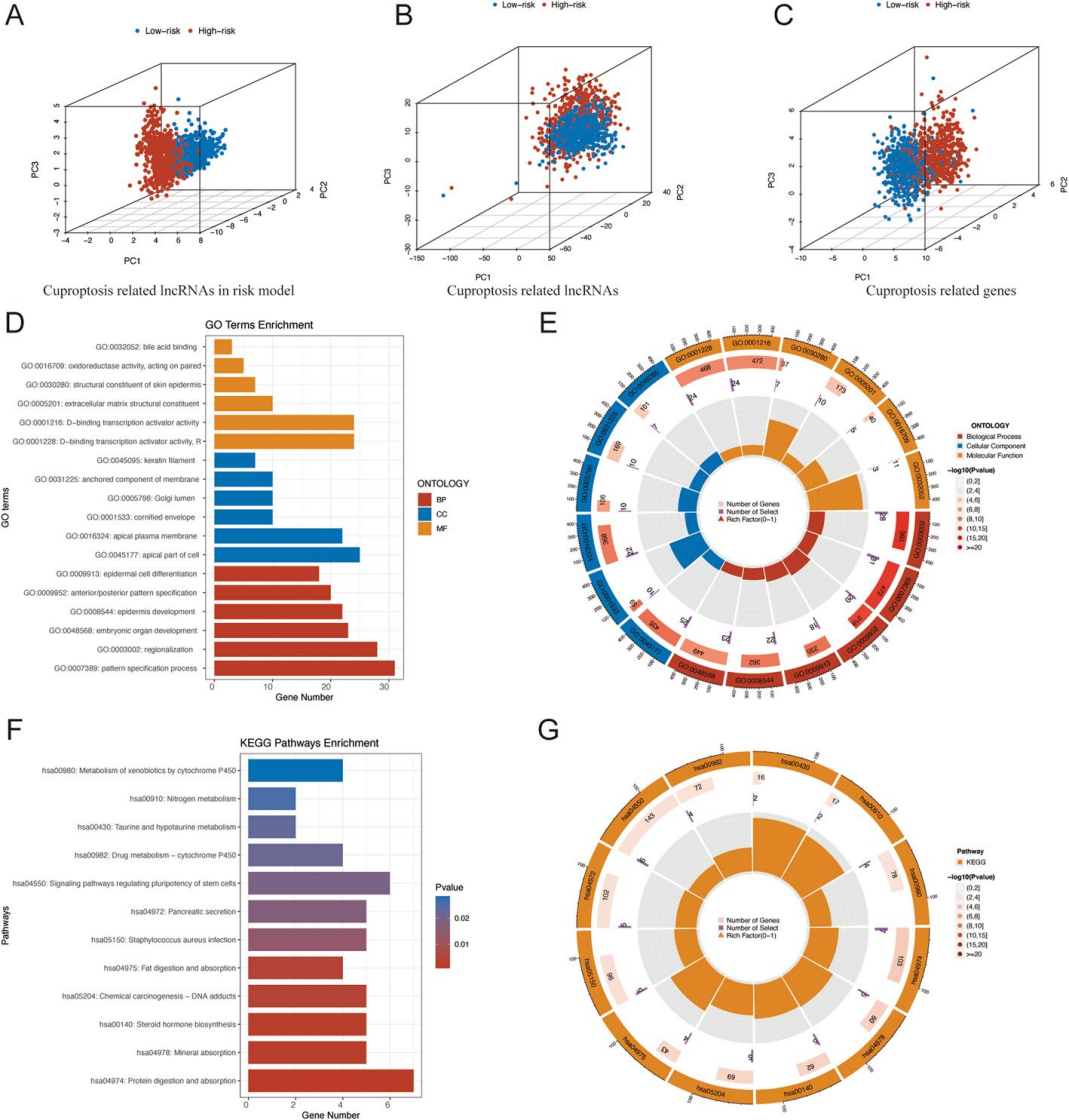
PCA, GO, and KEGG analyses. **A**–**C** 3D scatter plots of the sample distribution. **D**, **E** GO analysis of biological processes, cellular components, and molecular functions. **F**, **G** KEGG analysis of different signaling pathways

PCA was used to analyze the aggregation characteristics of the low-risk and high-risk sets. The outcomes presented that risk lncRNAs had superior classification capacity than all genes, cuproptosis regulators, and cuproptosis-related lncRNAs (Fig. 6A–C).

DEGs with the cut-off criteria of log2|FC|> 1 and FDR < 0.05 were chosen to further investigate the variations in biological processes and signaling pathways in the two groups. The GO analysis indicated that DEGs were involved in epidermal cell differentiation, anterior/posterior pattern specification, epidermis development, embryonic organ development, regionalization, and pattern specification process in the biological process (BP) category. The apical part of cell, cornified envelope, apical plasma membrane, Golgi lumen, anchored component of membrane, and keratin filament were enriched in DEGs at the cell component (CC) category. Besides, DEGs were mainly correlated with the bile acid binding, oxidoreductase activity, structural constituent of skin epidermis, extracellular matrix structural constituent, and DNA-binding transcription activator activity for the molecular function (MF) category (Fig. 6D, E; Additional file 3: Table S3).

In the KEGG analysis, these DEGs presented more enrichment in signaling pathways regulating pluripotency of stem cells, pancreatic secretion, staphylococcus aureus infection, steroid hormone biosynthesis, and protein digestion and absorption (Fig. 6F, G; Additional file 4: Table S4).

To compare the variations in biological processes and pathways between the high-risk and low-risk categories, we further conducted GSEA analyses (Additional file 5: Figs. S2, S3). The findings showed that the low-risk group had higher levels of base excision repair, citrate cycle, TCA cycle, oxidative phosphorylation, peroxisome, and ribosome. In the high-risk group, linoleic acid metabolism was enhanced.

### Correlation analysis between risk scores and gene mutations

**Fig. 7:**
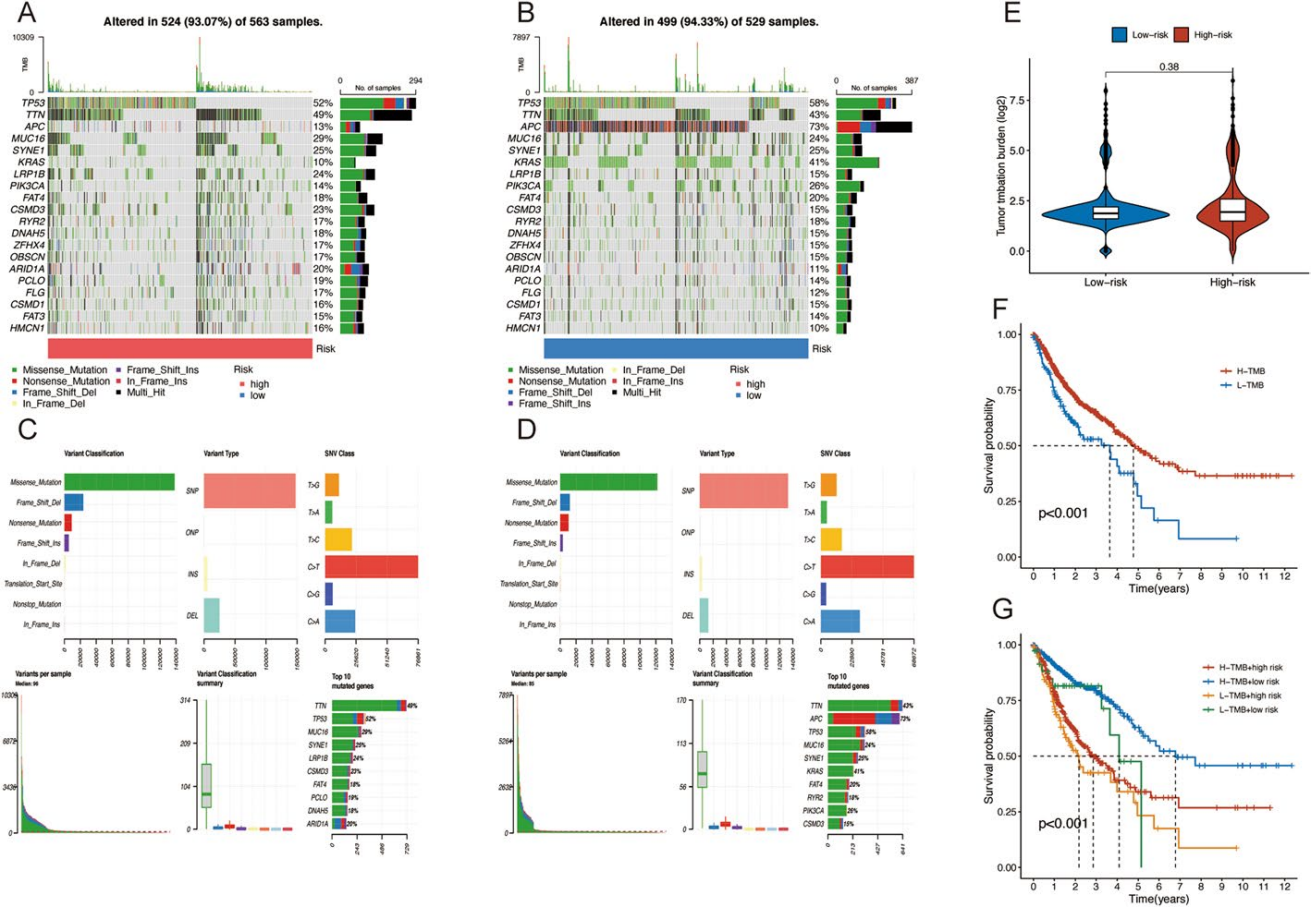
Somatic mutation analysis. Waterfall plots (**A**, **B**) and MAF-summary plots (**C**, **D**) of the somatic mutation profiles of two risk cohorts. **E** Comparison of the TMB level in the two cohorts. **F** Kaplan–Meier survival curves in the high-TMB group and low-TMB group. **G** Kaplan–Meier survival curves among different subgroups

The somatic mutations between the two groups were contrasted. TP53, TTN, APC, MUC16, SYNE1, KRAS, LRP1B, PIK3CA, FAT4, and CSMD3 were the ten most frequently altered genes. The low-risk set had more frequent TP53, APC, and KRAS mutations (Fig. 7A, B). And the alternation of APC mutation frequency was the most significant (from 13% in the high-risk group to 73% in the low-risk group). Detailed information on somatic mutation was presented in Fig. 7C and D. Then the relationship between TMB and risk sets was investigated. The result indicated no significant difference in TMB in low-risk and high-risk sets (Fig. 7E). The ATM patients were then separated into low-mutation and high-mutation categories by median TMB. According to the Kaplan–Meier curves, the high-mutation samples presented better survival than the low-mutation group (*p* < 0.001) (Fig. 7F). When TMB and risk scores were combined to evaluate the prognosis, we discovered that patients with higher TMB in the low-risk fraction presented the best prognosis, whereas patients with lower TMB in the high-risk subset had the worst survival rate (Fig. 7G).

### Immune landscape of ATM patients

**Fig. 8:**
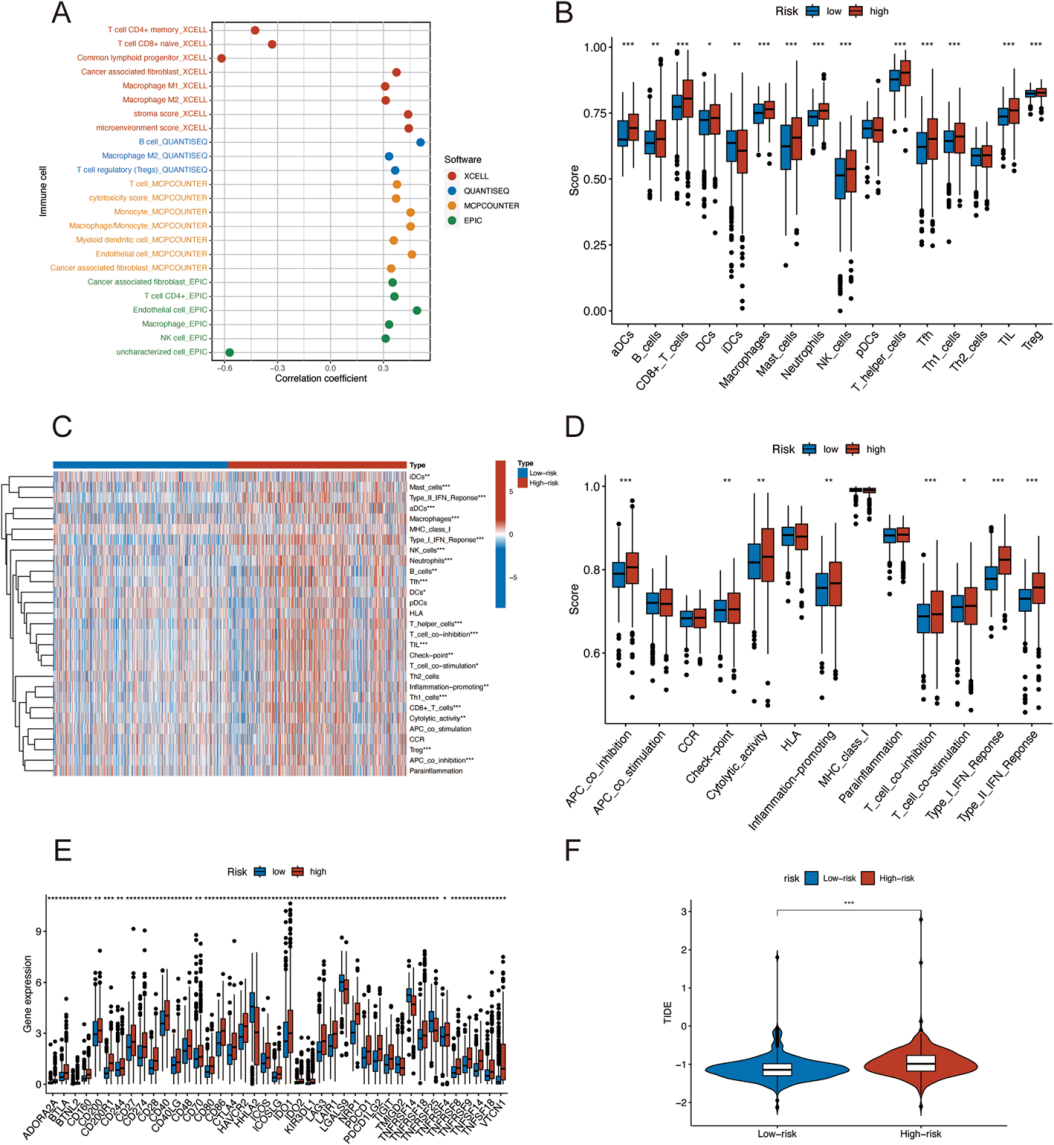
Differences of the immune landscape in two risk subsets of ATM. **A** Bubble plot of the correlation between the immune infiltration and risk scores via different algorithms. **B** Boxplot of the immune cell abundance. **C** Heatmap of the discrepancies of immune cell abundance via the ssGSEA method. **D** Boxplot for the immune-associated functions **E** Comparison of the immune checkpoints genes expression **F** TIDE scores. *p < 0.05, **p < 0.01, and ***p < 0.001; ns, no significance

Immune cell infiltration research revealed a link between risk scores and tumor-infiltrating immune cells. Immune cells were found to be closely related to high-risk ratings on various algorithms (Fig. 8A). According to the ssGSEA data, the high-risk group was abundant in immune cells such as aDCs, B cells, CD8 + T cells, DCs, macrophage, mast cells, neutrophils, NK cells, T helper cells, Tfh, Th1 cells, TIL, and Treg (Fig. 8B, C). Furthermore, immunological scores revealed that the immune function in the high-risk group was enriched, including APC co-inhibition, check-point, cytolytic activity, inflammation-promoting, T cell co-inhibition, type I IFN response, and type II IFN response (Fig. 8D).

Given the importance of ICIs in tumor treatment, we investigated immune checkpoint genes in both groups, indicating that major checkpoint gene expression differed between the two groups. And more immune checkpoint expression was observed in the high-risk set, such as CD27, CD40, CD86, LAG3, and NRP1 (Fig. 8E). TIDE scores in the high-risk group were considerably higher than in the low-risk group. This suggested that TIDE might be used to assess ATM patients' susceptibility to ICB therapy (Fig. 8F). Above all, these findings revealed that high-risk patients might be more sensitive to immunotherapy.

## Discussion

Despite a decrease in ATM morbidity, it continues to be the world's leading cause of mortality from malignant tumors today [2]. Thus, research into carcinogenesis and tumor growth pathways remains critical. However, most studies focus on a specific cancer type while providing little concrete information about the homogeneity of cancers. The common mechanisms of digestive tract tumorigenicity have been explored recently and present encouraging results [30, 31]. So, this study tried another view of the homogeneity of cancer development. According to Science, cuproptosis was a novel type of cell death, which played a critical role in the occurrence and development of ATM [3]. And the prognostic significance of cuproptosis-related lncRNAs in ATM hadn’t been investigated before. By using bioinformatics analysis, we identified novel biomarkers associated with clinical traits and common regulatory mechanisms of digestive tract cancers.

Many ATM patients are initially diagnosed in the middle or advanced stages. As a result, early diagnosis biomarkers and effective therapy techniques are essential for ATM patients. Numerous studies have shown that lncRNAs have an important role in the early detection of ATM. Li et al. conducted a meta-analysis of 40 original research articles involving 6,772 individuals, indicating that serum or plasma lncRNAs had high sensitivity and specificity in identifying gastric cancer [32]. Cheng et al. discovered that the lncRNA LINC00662 affected CLDN8/IL22 co-expression and stimulated the ERK signaling pathway to enhance colon cancer growth and metastasis [33]. Huang et al. demonstrated that the lncRNA-encoded peptide HOXB-AS3 inhibited the growth of colon cancer cells [34]. Roohinejad et al. found that PVT1 and CCAT1 lncRNAs were great markers for the early diagnosis of ESCC, which played a critical role in cancer cell growth and regulation [35].

Cuproptosis, a novel mode of cell death, was recently described. It was produced by excessive copper binding to lipoylated components of the tricarboxylic acid (TCA) cycle, leading to toxic protein stress and cell death [3, 36]. Researchers discovered that cuproptosis might play an important role in tumor proliferation, metastasis, and angiogenesis [5]. Nonetheless, the prognostic significance of cuproptosis-related lncRNAs in ATM is little understood. The goal of this work was to create a new cuproptosis-related lncRNAs profile to predict survival and tumor immunity in ATM patients.

We started by downloading clinical data, transcriptome sequencing data, and survival data on ATM patients from the TCGA and GEO databases. Then seven risk lncRNAs were identified and used for signature construction. AC016737.1, GK-IT1, LINC01555, SETBP1-DT, and SNHG4 were proven to play critical roles in multiple malignancies [37–41]. The genes AC048344.4 and AL590483.4 were undocumented, and it was currently unknown what the underlying mechanism of these lncRNAs in ATM was. These discovered lncRNAs were intriguing targets for cancer therapy and might very well contribute to the mechanism of ATM.

The risk value produced by the model, just as some recognized prognostic indicators like pathologic stages, could be used to independently predict the prognosis of ATM patients. Additionally, the risk model was more effective in predicting patient outcomes because it comprised only seven identified lncRNAs. The nomogram was subsequently approved as a technique for predicting the 1-, 3-, and 5-year OS of ATM patients. The congruence between the predictions made by the nomogram and the actual results was shown via calibration curves. Statistical investigation revealed that our prognostic signature was highly accurate and sensitive.

TMB has been shown to be an accurate predictor of the efficacy of immunotherapy [42, 43]. Then we analyzed the TMB landscape in both groups, and a higher level of TMB was discovered in the high-risk cohort. The results indicated that our signature might be used to identify individuals who might benefit from immunotherapy, potentially improving treatment results and reducing the risk of serious immune-related side events.

Numerous studies have shown that the tumor immunological microenvironment is critical in the formation and progression of ATM [44, 45]. Between the high-risk and low-risk groups in our investigation, the ssGSEA algorithm found a substantial difference in immune cell infiltration. These findings showed that cuproptosis was closely related to immune infiltration and the tumor-immune microenvironment in patients with ATM. A higher proportion of immune cells, such as aDCs, B cells, CD8 + T cells, DCs, macrophages, mast cells, neutrophils, NK cells, T helper cells, Tfh, Th1 cells, TIL, and Treg, were infiltrated in the high-risk fraction compared to the low-risk group. And patients in the low-risk group survived longer than those in the high-risk fraction. The findings suggested that worse survival outcomes for ATM patients were predicted by the enrichment of these immune cells.

Immune checkpoint inhibitors have ushered in a revolutionary age of cancer immunotherapy, with the potential to improve the treatment results of cancer patients [46]. Therefore, we investigated the differences in immune checkpoint gene expression between the high-risk and low-risk groups. According to the findings, immunological checkpoints such as CD27, CD40, CD86, LAG3, and NRP1, were more active in the high-risk group. Previous studies had partly explained the role of these immune checkpoints in ATM patients. For example, Rhyner et al. reported that the LAG3 expression on tumor-infiltrating lymphocytes was significantly associated with prognosis. LAG3 testing might aid in predicting outcomes for colon cancer patients and might help to find those who would benefit from adjuvant chemotherapy [47]. Additionally, gastric cancer and esophageal cancer were tightly linked to LAG3, which was thought to be a prospective therapeutic target for antitumor therapy [44, 48]. Above all, our signature suggested that for ATM patients at higher risk, medicines targeting these immune checkpoints would offer a viable therapy option.

Several studies indicated the role of copper in ATM. For instance, disulfiram (DSF)/Cu-induced formation of reactive oxygen species (ROS) resulted in the growth inhibition of GC cells via glycolysis [8]. Besides, Hu et al. found that copper could induce autophagic cell death by targeting ULK1 in colorectal cancer [49]. Based on the GO and KEGG analysis, we reasonably guessed that cuproptosis might be involved in ATM progression by influencing the activity of apical plasma membrane and D-binding transcription activator. And signaling pathways regulating pluripotency of stem cells and metabolism of xenobiotics by cytochrome P450 might be involved in pathway mechanism in ATM development. According to the GSEA analysis, the signaling pathway of the citrate cycle TCA cycle was enriched in the low-risk group. Cuproptosis occurs when copper binds to lipoylated enzymes in the TCA cycle, causing protein aggregation, proteotoxic stress, and cell death. As a result, the TCA cycle might be a critical route in ATM copper-dependent cell death. These findings suggested inhibiting the TCA cycle pathway could have anti-cancer effects in ATM. Nonetheless, these findings required additional investigation.

The advancement of interaction prediction research in various fields of computational biology has provided valuable insights into genetic markers and molecular mechanisms [50, 51]. At present, the interactions between lncRNA and miRNA are mainly obtained through biological experiments, but such experiments are often time-consuming and labor-intensive, it is necessary to design a computational method that can predict the interactions between lncRNA and miRNA. Wang et al. proposed a method based on graph convolutional neural and conditional random field for predicting lncRNA–miRNA interactions, which had an AUC value of 0.947 and presented higher prediction accuracy than the other methods [52]. Zhang et al. used network distance analysis to predict lncRNA–miRNA interaction, verifying the reliability of this method [53]. Besides, Liu et al. established a novel matrix factorization model to predict lncRNA–miRNA interactions, and the model obtained reliable performance [54]. Thus, the interaction prediction between cuproptosis-related lncRNAs and miRNA could be investigated by computational biology methods in the future.

### Limitation

Undoubtedly, our current research also included several limitations and flaws. First, the whole samples used for our signatures were obtained from the TCGA and GEO databases. Second, there weren’t enough clinical samples used to validate signatures. As a result, additional study in the following clinical stage is required. Finally, in vivo and in vitro investigations should be conducted to investigate the underlying processes of how these cuproptosis-related lncRNAs influence ATM.

## Conclusion

In summary, we effectively developed a novel seven lncRNAs signature with excellent sensitivities and specificities to predict survival outcomes in patients with ATM. Furthermore, our research sheds light on how cuproptosis-related lncRNAs in ATM function at the molecular level. The signature might offer direction for the customized treatment of ATM patients as well as aid in evaluating the effectiveness of targeted therapies and immunotherapy.

## Supplementary Information

**Additional file 1**. Cuproptosis genes.

**Additional file 2**. Univariate and multivariate Cox regression analysis in TCGA-ATM dataset.

**Additional file 3**. GO analysis of the DEGs.

**Additional file 4**. KEGG analysis of the DEGs.

**Additional file 5**. Supplementary Figures.

## Data Availability

The public datasets were obtained from TCGA (https://portal.gdc.cancer.gov/) and GEO (https://www.ncbi.nlm.nih.gov/geo/). GEO Accession Numbers: GSE40967, GSE53622, and GSE84437.
