# Supplementary material for: Identification of cuproptosis-related lncRNAs to predict prognosis and immune infiltration characteristics in alimentary tract malignancies
Source: BMC Bioinformatics. 2023 May 4;24:184. doi: 10.1186/s12859-023-05314-z (PMC10161432; doi:10.1186/s12859-023-05314-z)
Supplement: Supplementary file 2 — Additional file 2. Univariate and multivariate Cox regression analysis in TCGA-ATM dataset. [file 12859_2023_5314_MOESM2_ESM.docx]

Supplementary Table S2

Univariate and multivariate Cox regression analysis in TCGA-ATM dataset.

| Variable | Univariate analysis | | Multivariate analysis | |
| --- | --- | --- | --- | --- |
|  | HR | p value | HR | p value |
| Age | 1.39971196 | 0.00260649 | 1.79362018 | 0.0001811 |
| Gender | 1.43348618 | 0.00206275 | 1.39407147 | 0.06110866 |
| Grade | 1.36341726 | 0.01722325 | 1.26936614 | 0.08813578 |
| Stage | 1.7934702 | 1.89E-18 | 1.67015854 | 4.34E-07 |
| riskScore | 1.73225508 | 2.15E-24 | 1.31430199 | 0.0038704 |

HR, hazard ratio
