# Supplementary material for: Identification of cuproptosis-related lncRNAs to predict prognosis and immune infiltration characteristics in alimentary tract malignancies
Source: BMC Bioinformatics. 2023 May 4;24:184. doi: 10.1186/s12859-023-05314-z (PMC10161432; doi:10.1186/s12859-023-05314-z)
Supplement: Supplementary file 5 — Additional file 5. Supplementary Figures. [file 12859_2023_5314_MOESM5_ESM.docx]

****Supplementary Figures****


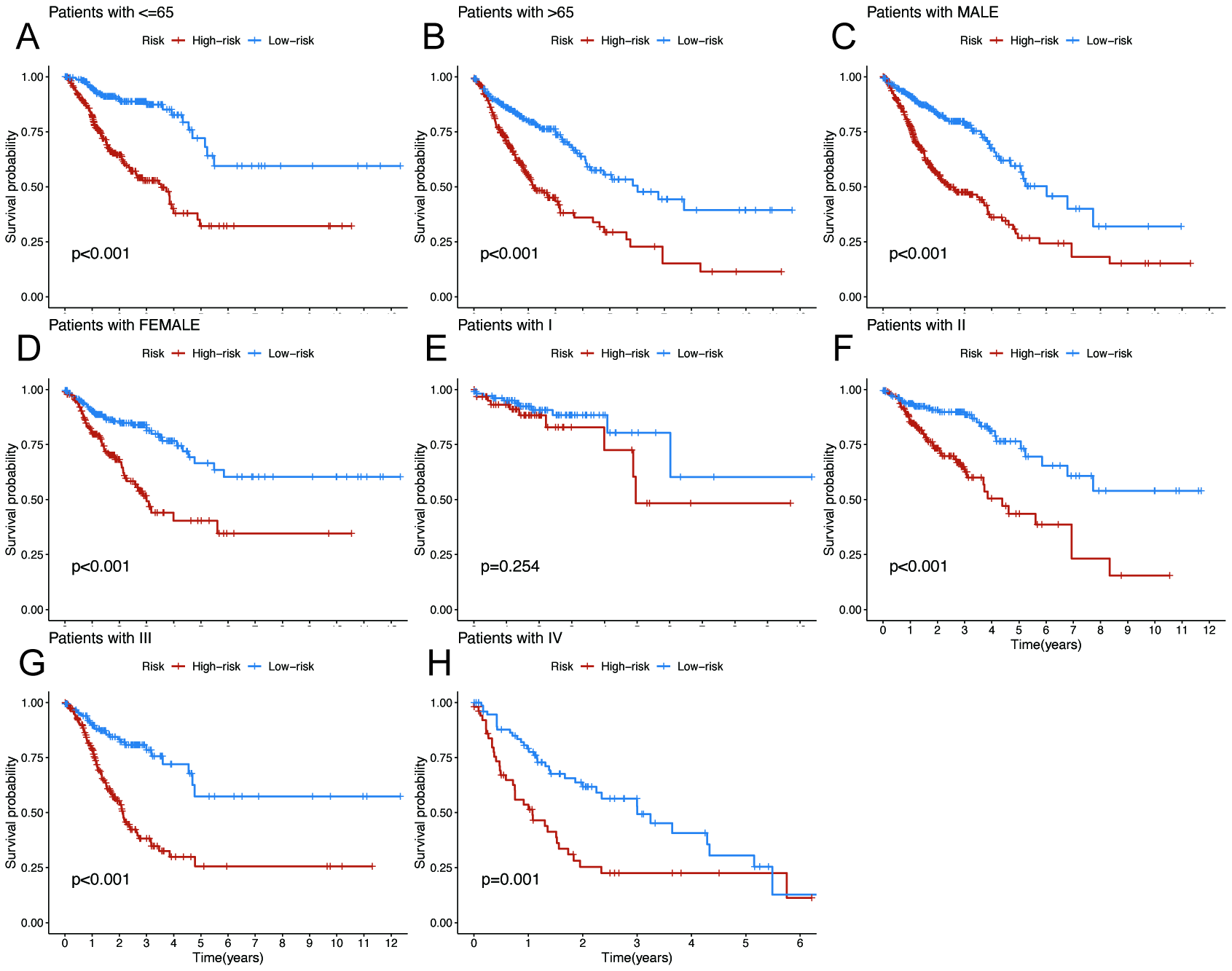


**Figure S1.** Kaplan–Meier survival curves of two risk cohorts in the subgroups based on ≤ 65 years of age (A), > 65 years of age (B), male sex (C), female sex (D), stage I (E), stage II (F), stage III (G), stage IV (H).


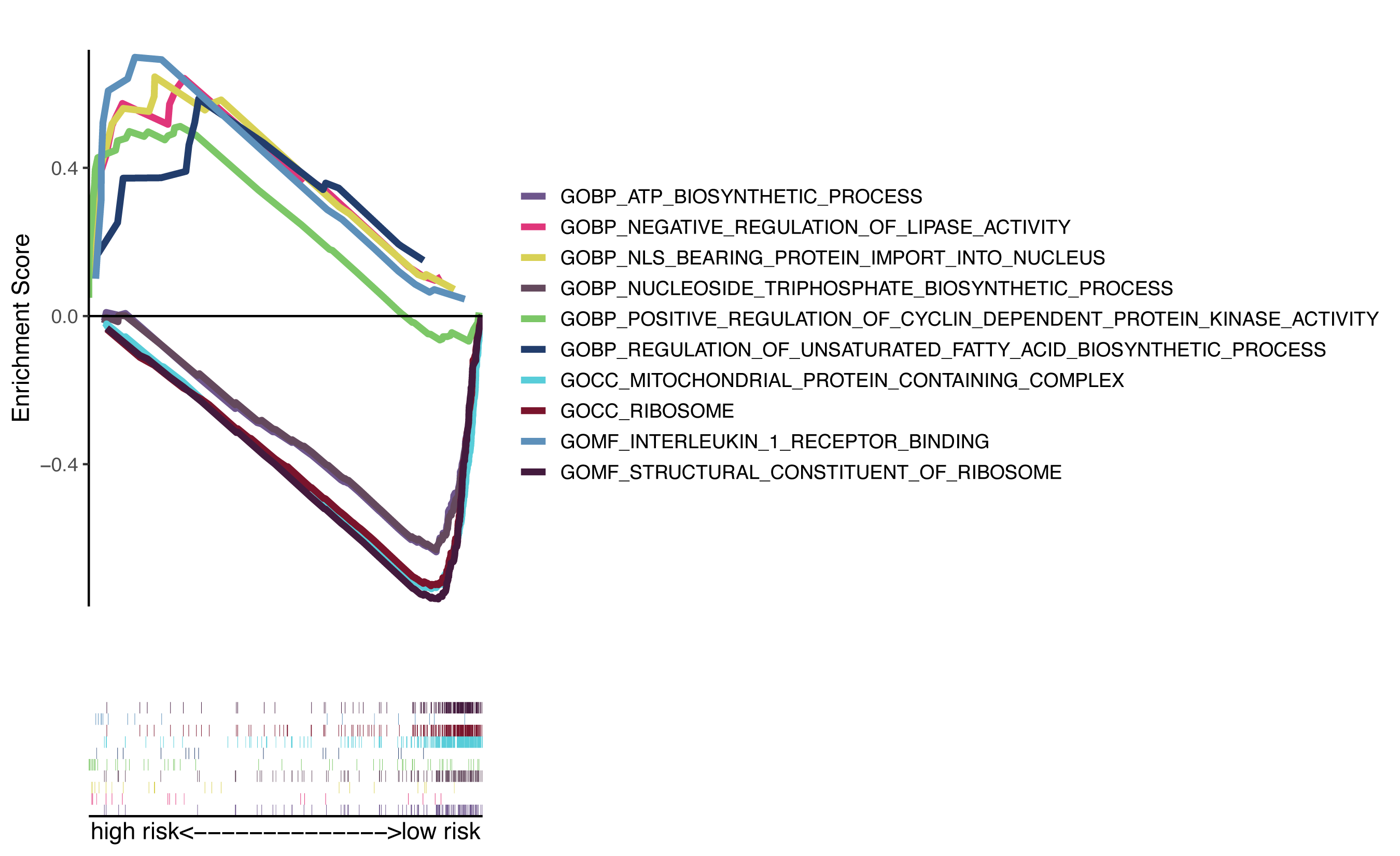


**Figure S2.** GSEA enrichment analysis of GO between the low- and high-risk groups.


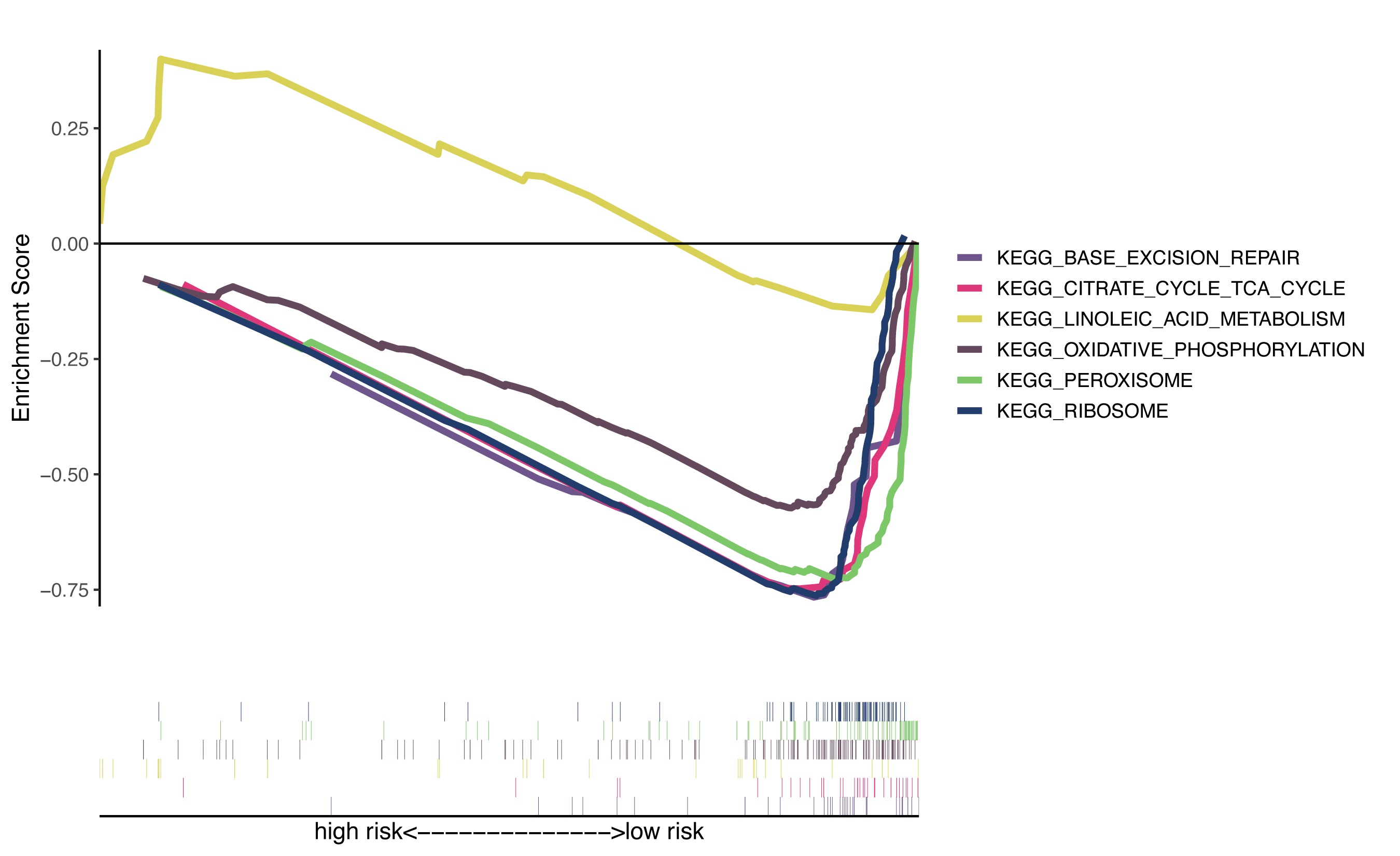


**Figure S3.** GSEA enrichment analysis of KEGG between the low- and high-risk groups.

****Supplementary Tables****

Table S1. Cuproptosis genes.

Table S2. Univariate and multivariate Cox regression analysis in TCGA-ATM dataset.

Table S3. GO analysis of the DEGs.

Table S4. KEGG analysis of the DEGs.
